# Using the Software DeepWings© to Classify Honey Bees across Europe through Wing Geometric Morphometrics

**DOI:** 10.3390/insects13121132

**Published:** 2022-12-08

**Authors:** Carlos Ariel Yadró García, Pedro João Rodrigues, Adam Tofilski, Dylan Elen, Grace P. McCormak, Andrzej Oleksa, Dora Henriques, Rustem Ilyasov, Anatoly Kartashev, Christian Bargain, Balser Fried, Maria Alice Pinto

**Affiliations:** 1 Centro de Investigação de Montanha, Instituto Politécnico de Bragança, Campus de Santa Apolónia, 5300-253 Bragança, Portugal; 2 Laboratório Associado para a Sustentabilidade e Tecnologia em Regiões de Montanha (SusTEC), Instituto Politécnico de Bragança, Campus de Santa Apolónia, 5300-253 Bragança, Portugal; 3 Research Center in Digitalization and Intelligent Robotics (CeDRI), Instituto Politécnico de Bragança, Campus de Santa Apolónia, 5300-253 Bragança, Portugal; 4 Department of Zoology and Animal Welfare, University of Agriculture in Krakow, 31-425 Krakow, Poland; 5 Department of Molecular Ecology & Evolution, School of Natural Sciences, Bangor University, Bangor, Gwynedd LL57 2DG, UK; 6 Taskforce Research, ZwarteBij.org VZW, 9890 Gavere, Belgium; 7 Zoology, Earth and Life Sciences, School of Natural Sciences, University of Galway, H91 TK33 Galway, Ireland; 8 Department of Genetics, Faculty of Biological Sciences, Kazimierz Wielki University, Powstańców Wielkopolskich, 85-090 Bydgoszcz, Poland; 9 Transplantology and Genotyping, Department of the Progressive Technologies, Bashkir State Agrarian University, 450001 Ufa, Russia; 10 Independent Researcher, 188338 Novosiverskaya, Russia; 11 Association pour la Sauvegarde de l’Abeillee Noire, 56069 Ile de Groix, France; 12 Swiss Association of Mellifera Bee Friends, mellifera.ch, Ahornstrasse 7, 9533 Kirchberg, Switzerland

**Keywords:** *Apis mellifera* subspecies, wing geometric morphometrics, honey bee classification, honey bee conservation, introgression

## Abstract

**Simple Summary:**

Wing venation traits are used to identify honey bee subspecies. While several wing-based tools are available, they suffer from weaknesses that were addressed by the recently developed software DeepWings©. This software allows fully automated identification of wing images in a friendly, free, and rapid manner. Here, we sought to test DeepWings© on 14,816 wing images representing 2601 colonies sampled in the native areas of three widespread subspecies in Europe: the Iberian honey bee (*Apis mellifera iberiensis*), the dark honey bee (*Apis mellifera mellifera*), both belonging to the M lineage, and the Carniolan honey bee (*Apis mellifera carnica*), belonging to the C lineage. DeepWings© classification of these colonies largely matched the endemic M and C lineages, with proportions of 71.4% and 97.6%, respectively. At the subspecies-level the matching proportions were 89.7% for the Iberian honey bee, 41.1% for the dark honey bee and 88.3% for the Carniolan honey bee, which can be explained by DeepWings© sometimes confounding closely related subspecies and, more importantly, by genetic pollution. A comparison between DeepWings© data and molecular data revealed that the agreement between the two is weaker when there is genetic pollution. Our results suggest that DeepWings© is a valuable tool for honey bee identification, which can be used not only for breeding and conservation but also for research purposes.

**Abstract:**

DeepWings© is a software that uses machine learning to automatically classify honey bee subspecies by wing geometric morphometrics. Here, we tested the five subspecies classifier (*A. m. carnica*, *Apis mellifera caucasia*, *A. m. iberiensis*, *Apis mellifera ligustica,* and *A. m. mellifera*) of DeepWings© on 14,816 wing images with variable quality and acquired by different beekeepers and researchers. These images represented 2601 colonies from the native ranges of the M-lineage *A. m. iberiensis* and *A. m. mellifera*, and the C-lineage *A. m. carnica*. In the *A. m. iberiensis* range, 92.6% of the colonies matched this subspecies, with a high median probability (0.919). In the Azores, where the Iberian subspecies was historically introduced, a lower proportion (85.7%) and probability (0.842) were observed. In the *A. m mellifera* range, only 41.1 % of the colonies matched this subspecies, which is compatible with a history of C-derived introgression. Yet, these colonies were classified with the highest probability (0.994) of the three subspecies. In the *A. m. carnica* range, 88.3% of the colonies matched this subspecies, with a probability of 0.984. The association between wing and molecular markers, assessed for 1214 colonies from the M-lineage range, was highly significant but not strong (r = 0.31, *p* < 0.0001). The agreement between the markers was influenced by C-derived introgression, with the best results obtained for colonies with high genetic integrity. This study indicates the good performance of DeepWings© on a realistic wing image dataset.

## 1. Introduction

The honey bee, *Apis mellifera* L. (Hymenoptera: Apidae), differentiated into over 30 subspecies [1,2,3,4,5], which belong to four main evolutionary lineages native to (i) western and north-eastern Europe, and north-western China (lineage M), (ii) central and south-eastern Europe (lineage C), (iii) Africa (lineage A), and (iv) the Near East and Central Asia (lineage O). Europe is the cradle of 10 such subspecies, of which eight belong to the M and C lineages. While the European M lineage comprises only *A. m. mellifera* and *A. m. iberiensis*, it spreads across a wider geographical and climatically more diverse area than the other six subspecies of C-lineage ancestry. This area extends from the Iberian Peninsula to southern Scandinavia and from Britain and Ireland to the Ural Mountains [5]. In contrast, the native area of the six C-lineage subspecies is limited to the Apennine and Balkan peninsulas, bordered at the north by the Alps and the Carpathians, and at the south by Sicily and the west Aegean islands [5]. Remarkably, despite the greater potential of the M lineage to adapt to more extreme environments, the C lineage includes two of the three subspecies favored in apiculture: *A. m. carnica* and *A. m. ligustica*. These, together with the O-lineage *A. m. caucasia*, were introduced worldwide due to their perceived gentle behavior and high productivity [5,6,7,8,9,10]. As a result, in many places of the M-lineage distributional range, particularly north of the Pyrenees, the genetic integrity of the native *A. m. mellifera* was threatened by gene flow from those foreign subspecies [9,10,11,12,13,14]. In an attempt to restore and protect the *A. m. mellifera* gene pool, conservation efforts sprouted in Europe [15] and an association (SICAMM–Societas Internationalis pro Conservatione *Apis melliferae melliferae*) for its protection was founded in 1994. These efforts require tools to identify colonies before they can be moved to conservation areas or to monitor the efficiency of isolated mating stations. On the other hand, identification tools may also be useful to C-lineage queen breeders in Eastern and Southeastern Europe.

The molecular tool kit for honey bee identification includes different markers of the mitochondrial (e.g., tRNAleu-cox 2 intergenic region) and nuclear DNA (e.g., microsatellites and single nucleotide polymorphisms [SNPs]) [16,17,18,19,20,21,22,23,24,25]. Although this toolkit proved to be powerful for honey bee identification [25,26,27,28,29,30,31,32,33], its use by beekeepers is very limited [34]. This is because molecular methods require trained personnel in addition to costly equipment and reagents, making genetic analysis of colonies unaffordable to beekeepers. In contrast, morphometric methods are cost-effective, need only a microscope with an attached camera, and, when based on wing traits, can more easily be implemented by beekeepers [23,35].

Subspecies identification by classical morphometry comprises 42 characters, including the size of anatomical structures, such as proboscis, femur, tergite, and sternites; discrete classes of pigmentation; length and width of wings, and angles in wing venation [5]. However, the manual measuring of this full-body set is very time-consuming and only a subset of wing characters proved to be informative for subspecies discrimination [23]. One of the most intensive assessments of wing shape variation is provided by the Discriminant Analysis with Numerical Output (DAWINO) method. This method is based on 30 characters extracted from vein lengths, their ratios, and vein angles. On the other side of the spectrum are the methods that only require estimations of the Cubital Index (CI), Hantel Index (HI), and/or Discoidal Shift Angle (DSA). These are popular amongst many beekeepers involved in the conservation of *A. m. mellifera* [34] as they are simple and can be implemented by semi-automatic software, such as BeeMorph (http://www.hockerley.plus.com/ accessed on 1 July 2022 ) or CBeeWings (http://www.cybis.se/cbeewing/ accessed on 1 July 2022). The problem is that these software packages provide a less reliable identification than that obtained from character-intensive methods. In addition, they require manual annotation of the vein junctions from which the indexes are calculated, which is time-consuming and prone to error. 

Another way of assessing wing shape variation is through wing geometric morphometrics (WGM). This method is recognized as robust and reliable in insect taxonomy and is widely used in honey bee subspecies identification for varying purposes, including conservation [36,37,38,39,40,41,42]. WGM uses the coordinates defined by 19 landmarks located in the vein junctions to capture variation in wing shape [43] and can be implemented by the software IdentiFly [36]. However, IdentiFly is a semi-automatic tool that requires several steps before the wings can be fully identified, which makes its use difficult for the layman. The most recent advance in WGM uses deep learning to automatically extract the 19 landmarks from the right forewing of honey bee workers [44]. The approach is implemented by the software DeepWings©, which allows fully automated identification of honey bees. DeepWings© is very friendly, requiring only that the users drag the wing images into a file drop zone. Then, for each analyzed wing, it automatically retrieves the classification probabilities for the top three subspecies and the estimation of CI, HI, and DSA along with the landmark coordinates. DeepWings© is offered as a free web service at https://deepwings.ipb.pt. (accessed on 1 November 2022)

Herein, we employed DeepWings© to identify 2601 colonies from the analysis of 14,816 wings. These colonies were located in 15 countries, covering the native ranges of *A. m. iberiensis*, *A. m. mellifera* and *A. m. carnica*. In addition, we compared the wing shape data with molecular data for a subset of *A. m. iberiensis* and *A. m. mellifera* colonies. Our objectives were (i) to evaluate the functionality of DeepWings© when processing a massive number of wing images of varying quality and produced by different persons; (ii) to assess how closely the colonies identified by DeepWings© matched the endemic subspecies distribution, with an emphasis on M-lineage subspecies; and (iii) to assess the association between the identification produced by DeepWings© and that inferred from molecular markers.

## 2. Materials and Methods

### 2.1. Wing Samples

**Figure 1:**
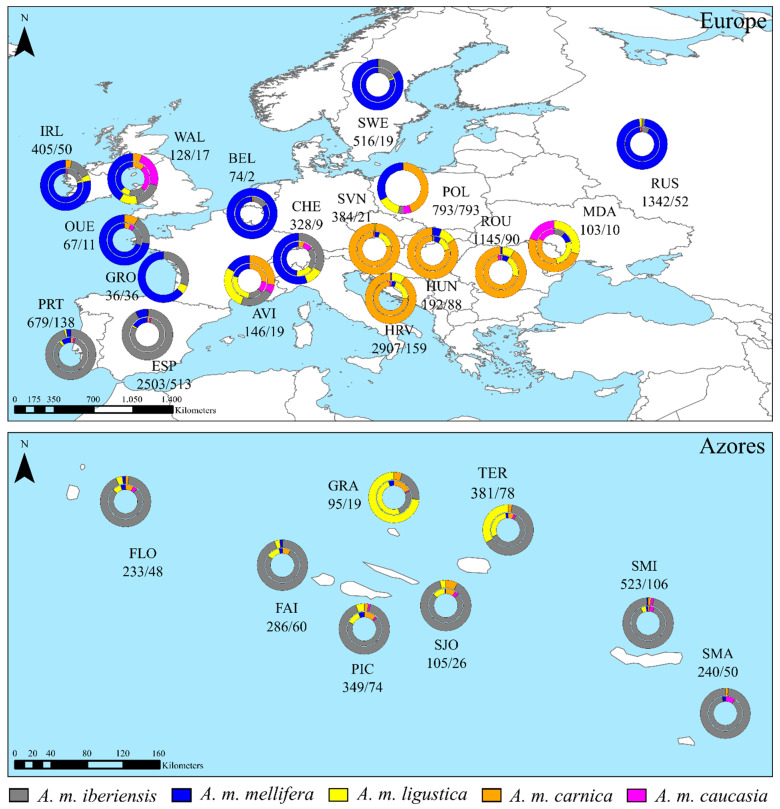
DeepWings© classification at the individual wing and colony levels. Sections in each donut chart represent the proportion of wings (inner ring) or colonies (outer ring) classified into each subspecies. Sample sizes of individual wings/colonies are indicated for each location. In the cases of Groix (France), Poland, and Hungary, each colony is represented by a single wing, so that classification for wings and colonies is coincident. AVI-Avignon, BEL-Belgium, CHE-Switzerland, ESP-Spain, FAI-Faial, FLO-Flores, GRA-Graciosa, GRO-Groix, HRV-Croatia, HUN-Hungary, IRL-Ireland, MDA-Moldova, OUE-Ouessant, PIC-Pico, POL-Poland, PRT-Portugal, ROU-Romania, RUS-Russia, SJO-São Jorge, SMA-Santa Maria, SMI-São Miguel, SVN-Slovenia, SWE-Sweden, TER-Terceira, and WAL-Wales.

A total of 14,816 right forewing images of workers were obtained from 2601 colonies located in 15 countries (Figure 1, Table S1). The sampling effort encompassed the native distribution of the (i) M-lineage subspecies *A. m. iberiensis* (Portugal, Spain, and historical introduction in the Azores), *A. m. mellifera* (Belgium, France, Ireland, Poland, Russia, Sweden, Switzerland, UK), and (ii) C-lineage subspecies *A. m. carnica* (Croatia, Hungary, Moldavia, Romania, and Slovenia). Samples were collected from hives, except for Hungary and Poland, where the great majority were collected from flowers. However, these samples most likely represent independent colonies, given the > 3 km distance between sampling locations. Some of the samples of *A. m. mellifera* were collected from protected apiaries (Table S1). The number of wings per colony varied between one (all samples collected from flowers and samples from Groix) and 39, with a median of 5. Most wings were photographed using a stereomicroscope attached to a digital camera, with variable quality and dpi resolution (Figure S1).

### 2.2. DeepWings© Analysis

Given that the samples were collected from the native ranges of *A. m. iberiensis*, *A. m. mellifera*, and *A. m. carnica*, wing images were classified using the five subspecies classifier of DeepWings© [44], as it is more accurate than the 26 subspecies classifier. Wing images were entered for each colony in batches varying between 4 and 39, except for samples from Groix, Poland, and Hungary. For these locations, because there was only one wing per colony, 40 images (the maximum accepted by the program) were loaded simultaneously.

The output of DeepWings© used in this study included lineage and subspecies classification. The five-subspecies classification model of DeepWings© predicts the probability that a given wing belongs to *A. m. iberiensis*, *A. m. mellifera*, *A. m. carnica*, *A. m. ligustica*, or *A. m. caucasia* and corresponding lineage. The software retrieves the three highest prediction probabilities for each wing individually and for an average wing estimated from the wing batch dragged into the file drop zone. DeepWings© constructs the average wing by averaging the coordinates of each of the 19 landmarks across all the wings processed in one batch [44]. When all the wings from a colony are simultaneously uploaded, as is carried out here, the estimated average wing represents the colony and the classification at the colony level can be retrieved for the average wing. We used the wings output at both individual and colony levels. The wings were assigned to one of the five subspecies based on the highest prediction probability, even if the probability was low.

### 2.3. Association between Wing Data and Molecular Data

The association between DeepWings© classification and that obtained from molecular markers (microsatellites and SNPs) was assessed for 1214 colonies sampled from the native ranges of *A. m. iberiensis* (Portugal, Spain) and *A. m. mellifera* (France, Ireland, Wales, Russia). For each colony, the highest prediction probability of belonging to M lineage, as inferred from the 19 landmark coordinates using DeepWings©, was compared against the corresponding M-lineage membership proportion, as inferred from different sets of microsatellites or SNPs (Table S1) using the software Structure [45] or Admixture [46], respectively. The molecular dataset was generated in previous works (see Table S1). 

### 2.4. Statistical Analysis

The probability data obtained from the average wing for the subspecies identified by DeepWings© did not follow a normal distribution, as per the Kolmogorov–Smirnov test. Accordingly, the summary statistics were presented for each location as medians and interquartile ranges (median, interquartile range [IQR]; Tables S2 and S3). The distributions of the probability data points were compared among the identified subspecies in each dataset using the Mann–Whitney U test or the Kruskal–Wallis test, followed by the Dunn’s multiple comparison test with statistical significance levels (*p*) adjusted by Bonferroni. The association between the probability of belonging to the M lineage, as inferred by DeepWings© from wing shape data, and the membership proportion in the M lineage, as inferred from microsatellite or SNP data, was assessed using the Spearman’s rank-order correlation coefficient (r). All statistical tests were conducted on Graph Pad Prism version 5.01 for Windows, GraphPad Software, San Diego, CA, USA.

## 3. Results

### 3.1. Classification of the Total Wings Dataset

A total of 14,816 worker forewings, representing 2601 colonies, were processed by DeepWings©. From these, 856 (5.8%) were rejected by the software as the 19 landmarks could not be annotated due to different image problems (Figure S1). The rejected subset included 106 wings from Poland and 7 from Hungary. Since the colonies of these two countries were mostly represented by single wings, the total number of classified colonies decreased to 2488. In 82.8% (709) of the discarded wings, rejection was mainly due to the very low resolution of the images and noisy background, leading DeepWings© to aggregate close landmarks. The remaining 147 (17.2%) images displayed some kind of corruption, including missing landmarks (9, 1.1%), folded or twisted wings (9, 1.1%), presence of artifacts on the images (37, 4.3%), overlapping wings (43, 5.0%), or broken wings with missing landmarks (49, 5.7%). The proportion of rejected wings varied among datasets, ranging from 0.0% (Portugal, 0 wings) to 39.1% (Ouessant, France, 43 wings). This meant an average (± SD) success in automatic landmark annotation of 92.23% ± 9.36 across the individual datasets.

The final sample sizes identified by the five-subspecies classifier of DeepWings© were 13,960 for wings and 2488 for colonies. Each wing was classified into the subspecies that showed the highest prediction probability, ranging from as low as 0.300 to 1.000, with a median of 0.968. The classification results at the colony level (inferred from the average wing) were similar, as the highest probability ranged from 0.309 to 1.000, with a median of 0.944. The highest median probability was obtained for wings identified as *A. m. mellifera* (median probability = 0.999, IQR = 0.023) and the lowest for wings identified as *A. m. caucasia* (0.702, 0.339). Analysis of the 2488 colonies further confirmed this pattern, with *A. m. mellifera* reaching a median of 0.994 (0.075) and *A. m. caucasia* 0.636 (0.334). This result makes sense, as colonies were not sampled in the native distribution of the Caucasian subspecies.

Table 1 shows the percentages of wings and colonies, sampled within the range of *A. m. iberiensis*, *A. m. mellifera*, and *A. m. carnica*, for the top two probabilities and for an arbitrary 0.950 probability threshold. For the sake of this table presentation, all the colonies from the Azores were included in the range of *A. m. iberiensis,* as this subspecies was originally introduced by the Portuguese settlers in historical times [47]. Despite the hybrid zone reported in the southern part of Poland [5], Polish colonies were included in the range of *A. m. mellifera,* as the great majority of them originated from elsewhere. Finally, all the colonies from Romania were included in the range of *A. m. carnica,* as the other native subspecies of Romania, *A. m. macedonica* [5], is not represented in the five-subspecies classifier and the majority of colonies were sampled in the *A. m. carnica* native range. As shown in Table 1, most wings and colonies were assigned to the expected subspecies. The highest proportions were observed for *A. m. iberiensis* for both wings (77.2%) and colonies (89.7%) and the lowest for *A. m. mellifera* (wings: 67.1%; colonies: 41.1%). However, when the 0.950 threshold was applied, the highest proportions of wings (86.7%) and colonies (97.3%) assigned to the expected subspecies were obtained for *A. m. carnica* in its native range.

The classification proportions of wings and colonies calculated using the highest prediction probability are shown by country in Figure 1. As before, the majority of the individual wings and colonies met the expectations concerning the native range of subspecies or lineages. The classification of the individual wings did not completely match the classification inferred from the average wing for colonies, although the proportions were similar (Tables S2 and S3). However, often, the number of subspecies identified from individual wings was higher than that identified from colonies. For example, in Portugal, *A. m. carnica* (17 wings) and *A. m. caucasia* (13 wings) were only identified at the individual wing level. When the classification was conducted at the colony level, these two subspecies were no longer detected. Because colony-level classification is more meaningful for apiculture than individual-level classification, the following results will be presented only for colonies. Furthermore, the common practice of wing-based identification is to average out intra-colony variation through analysis of multiple wings per colony [25].

### 3.2. Classification of Colonies Sampled in the Native Range of A. m. iberiensis

**Figure 2:**
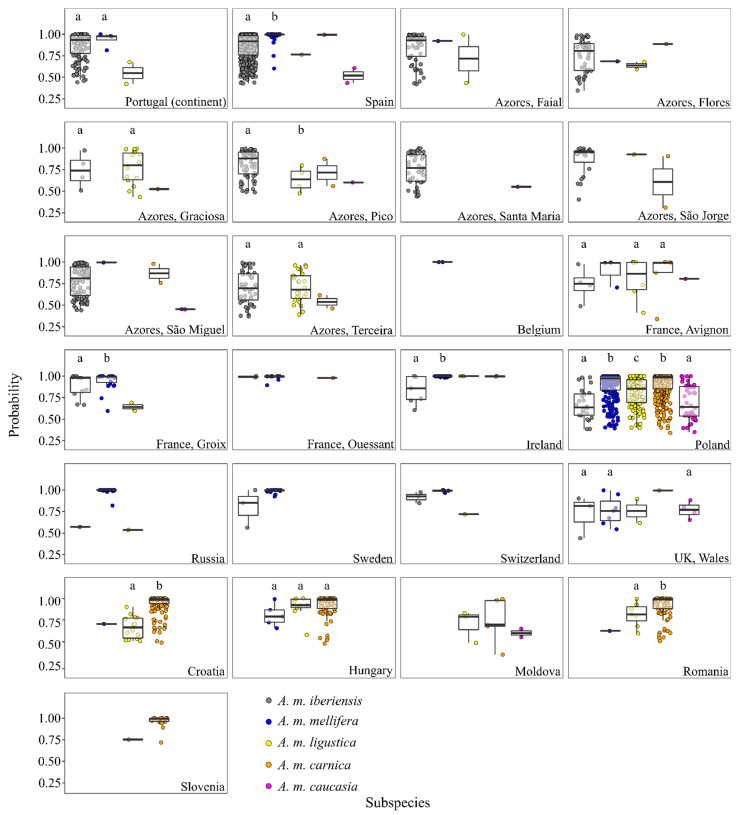
Classification probabilities calculated for colonies from the average wing with DeepWings©. Each dot represents a colony. Boxplots represent the median, interquartile range, maximum, and minimum. Groups with the same letter on top have similar distributions in the classification probabilities. Groups with different letters have different distributions in the classification probabilities for a significance level of 0.05.

Of the 651 colonies sampled in the *A. m. iberiensis* native range, 603 (92.6%) were classified as *A. m. iberiensis*, with a median probability of 0.919 (0.225). A higher proportion was found in Portugal (95.7%) than in Spain (91.8%), with median probabilities of 0.935 (0.210) and 0.918 (0.226), respectively (Table S3, Figure 2). The second most detected subspecies was *A. m. mellifera*, representing only 4 (2.9%) and 38 (7.4%) colonies in Portugal and Spain, with median probabilities of 0.976 (0.141) and 0.998 (0.005), respectively. While in Portugal, no differences were found in the distribution of the classification probabilities between the two M-lineage subspecies (U = 180.00, *p* > 0.05), in Spain, *A. m. mellifera* showed an unexpectedly higher median probability (U = 2490.00, *p* = 0.0001). *A. m. ligustica* was also detected in Iberia, although with a residual proportion (0.5%) and a low median probability (0.676, 0.340). *A. m. carnica* and *A. m. caucasia* colonies were detected exclusively in Spain, with the former representing only one colony (0.2%), with a probability of 0.994, and the latter representing two colonies (0.4%), with a median probability of 0.520 (0.172). When analyzed at the lineage level, nearly all colonies (99.1%) were assigned to the expected M-lineage.

In the Azores, *A. m. iberiensis* was also the most frequently identified subspecies on six of the eight sampled islands (Figure 1), although with a lower median probability (0.888, 0.274) than that found in mainland colonies (0.919, 0.225; Table S3). The highest median probability was observed for São Jorge (0.953, 0.210) and the lowest for Santa Maria (0.769, 0.318), where 23 (88.5%) and 49 (98.0%) colonies had an average wing shape closer to *A. m. iberiensis*, respectively. Only Graciosa and Terceira had a substantial proportion of C-lineage, with 73.7% and 33.3% of the colonies classified as *A. m. ligustica*. The median probability was higher for Graciosa (0.801, 0.349) than for Terceira (0.679, 0.288), but these were not significantly different from the median probabilities obtained for *A. m. iberiensis* in both islands (Graciosa: U = 25.00, *p* = 0.79; Terceira: U = 649.00, *p* = 1.00). A low proportion of *A. m. ligustica* (4 colonies, 5.4%) was also detected on Pico, although with a significantly (U = 50.00, *p* = 0.04) lower median probability (0.637, 0.280) than that obtained for *A. m. iberiensis* (0.881, 0.260).

### 3.3. Classification of Colonies Sampled in the Native Range of A. m. mellifera

Of the 1008 colonies sampled in the *A. m. mellifera* native range, 414 (41.1%) were classified as *A. m. mellifera*, with a median probability of 0.994 (0.082). Except for Avignon (France), Wales (UK), and Poland, the remaining locations had a high proportion of colonies classified as *A. m. mellifera* (Table S3, Figure 2). The two colonies from Belgium had wing shapes matching *A. m. mellifera*, with a high median probability (1.000, 0.000). In Russia, 50 (96.2%) colonies showed high classification probabilities for *A. m. mellifera* (0.998, 0.002), and only two were classified as *A. m. iberiensis* and *A. m. ligustica*, but with low probabilities: 0.571 and 0.537, respectively. A lower proportion of colonies from Ouessant (8, 72.7%), Groix (23, 63.9%), Ireland (39, 78.0%), Switzerland (5, 55.6%), and Sweden (16, 84.2%) were classified as *A. m. mellifera*, despite their high probabilities (0.991 ≤ median ≤ 1.000, 0.002 ≤ IQR ≤ 0.088). In these locations, *A. m. iberiensis* was the second most frequently identified subspecies, with a lower median probability in Ireland (U = 47.00, *p* = 0.005) and Groix (U = 73.00, *p* = 0.04). Therefore, when classified at the lineage level, most colonies (>93.0%) matched the expected M lineage (Figure 1). 

A high proportion of colonies from Avignon (63.2%), Wales (41.2%), and Poland (64.1%) had average wings more similar to subspecies of C- and O-lineage ancestries than to *A. m. mellifera* (Figure 1). In Poland, colonies were classified as *A. m. carnica* (44.5%) more frequently than as *A. m. ligustica* (14.1%) or *A. m. caucasia* (5.5%), with a significantly (0.53 < z < 7.07, 1.82 × 10^−11^ < *p* < 1.99 × 10^−6^) higher median probability of 0.983 (0.148) vs. 0.855 (0.268) or 0.641 (0.316), respectively. In contrast, in Wales, colonies classified as *A. m. caucasia* (23.5%) were more frequent than *A. m. ligustica* (11.8%) or *A. m. carnica* (5.9%).

### 3.4. Classification of Colonies Sampled in the Native Range of A. m. carnica

Of the 368 colonies sampled in the *A. m. carnica* native range, 325 (88.3%) were classified as *A. m. carnica*, with a median probability of 0.984 (0.089). The highest proportion was found in Slovenia (95.2%), with a median probability of 0.993 (0.040), followed by Romania (91.1%), with a median probability of 0.989 (0.117), Croatia (90.6%), with a median probability of 0.983 (0.058), Hungary (84.1%), with a median probability of 0.982, (0.121), and finally Moldova (50.0%), with the lowest median probability (0.695, IQR = 0.471), as shown in Table S3, Figure 2. Colonies classified as *A. m. ligustica* were also found in these five countries, but with lower proportions (4.8% for Slovenia, 7.8% for Romania, 8.8% for Croatia, 10.2% for Hungary, and 30.0% for Moldova) and significantly lower probabilities in Croatia (median = 0.664, IQR = 0.248; U = 122.00, and *p* = 6.27 × 10^−8^) and Romania (median = 0.815, IQR = 0.252; U = 134, and *p* = 0.020). In Hungary, the probabilities of colonies classified as *A. m. ligustica* (median = 0.922, IQR = 0.122) and *A. m. mellifera* (median = 0.790, IQR = 0.240) were similar to those of *A. m. carnica* (H(2) = 5.245, *p* = 0.072). Colonies classified as *A. m. mellifera* (1.9%) and *A. m. caucasia* (0.5%) were rare (Figure 1) and showed low median probabilities, varying between 0.600 (*A. m. caucasia* in Moldova) and 0.790 (*A. m. mellifera* in Hungary, Table S3).

### 3.5. Association between Wing Data and Molecular Data

**Figure 3:**
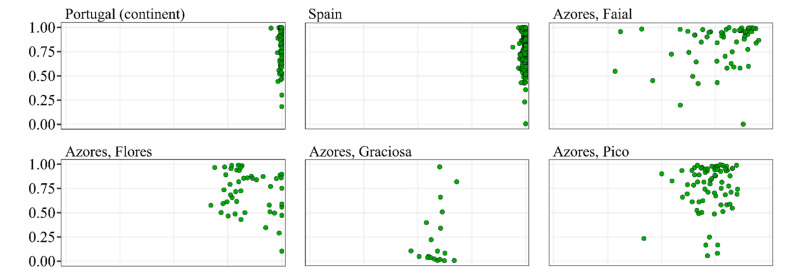

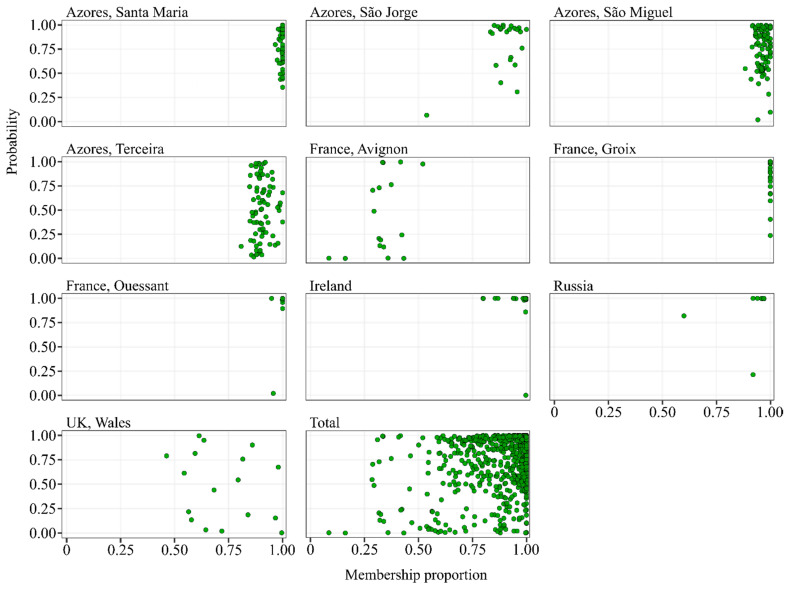
Scatter plots showing the probability of belonging to M lineage (Y-axis) vs. membership proportions in M lineage (X-axis) for individual datasets and for the whole dataset. Classification probabilities were inferred from wing images using the software DeepWings©, whereas membership proportions were inferred from microsatellites (Groix, Ireland and Russia) or SNPs (Portugal, Spain, Azores, Avignon, Ouessant, and Wales) using the software Structure or Admixture, respectively. Each dot represents a colony.

The association between the probability of belonging to the M lineage, as inferred by DeepWings© from wing shape data, and the membership proportion in the M lineage, as inferred from microsatellite or SNP data, is shown for all samples and locations in Figure 3. A good agreement between the morphological and molecular markers was observed for most of the samples from Spain, mainland Portugal, Santa Maria, São Miguel, São Jorge, Faial, Flores, Groix, Ouessant, Ireland, and Bashkortostan, as they lie in the upper quarter of both the X and Y-axis. This is not the case for the other locations, as samples are more scattered in the two-dimensional space, with many of them exhibiting high values for the molecular marker (X-axis) and low values for the morphological marker (Y-axis) or, less commonly, the opposite. For example, in the dataset of Terceira, 23.0% of the samples lie in the upper quarter for the molecular marker (>0.75) and in the lower quarter for the morphological marker (<25), indicating a poor agreement between the two. Nonetheless, for the whole dataset (N = 1214), a significant association was found between the two markers, as revealed by Spearman’s correlation test (r = 0.31; 0.25 < 95% confidence interval < 0.36; *p* < 0.0001).

## 4. Discussion

In this study, 14,816 wings representing 2601 colonies from 15 countries and covering the native ranges of *A. m. iberiensis*, *A. m. mellifera*, and *A. m. carnica* were analyzed using the WGM approach implemented by DeepWings©. This large and diverse dataset of wing images, originating from such a wide geographical range and acquired using varied image acquisition systems, offered a unique opportunity to test the performance of DeepWings© under real conditions. Moreover, the interaction with the numerous wing image contributors (beekeepers and researchers), who have different experiences and needs, allowed us to introduce several improvements in the software. These included (i) estimation of CI, HI, and DSA; (ii) display of the landmark-annotated wing images; (iii) production of a table with the landmark coordinates; (iv) inference of an average wing from a batch of wings, allowing classification of a colony from multiple wings; and (v) display of the three best classifications for the analyzed wings. DeepWings© successfully classified 94.2% of the wings, consistent with the rate predicted by the software developers [44].

The classification of the European colonies largely matched the endemic M and C-lineages, with proportions of 71.5% and 97.6%, respectively, as the top two probabilities were typically assigned to subspecies sharing lineage ancestry. However, when analyzed at the subspecies level, the matching proportions decreased, as samples collected in the *A. m. mellifera* range were often classified as *A. m. iberiensis* (the reverse was less frequent) and samples collected in the *A. m. carnica* range were often classified as *A. m. ligustica*. These findings are not surprising given that subspecies belonging to the same evolutionary lineage share a recent ancestor and are, therefore, genetically closely related [48]. Furthermore, European subspecies are largely parapatric and meet in natural contact zones where admixture occurs [28,30,49]. More importantly, due to beekeeping activities involving large-scale colony transhumance and queen trading, many subspecies belonging to the same or different lineages now occur in artificial sympatry, leading to further erosion of the boundaries between subspecies and to the breakdown of subspecies integrity [10,32,50,51,52,53]. These phenomena help explain the large dispersion of the probability values observed for the different locations, both within and between subspecies (Figure 2). Alternatively, but not mutually exclusive, DeepWings© is unable to recognize the full spectrum of natural variation, therefore failing the accurate classification of many colonies. The reference database used by the classification module of DeepWings© was constructed from a small subset of the original wings analyzed by Ruttner in his seminal taxonomic work [44]. Therefore, it only partially covers the natural variation in wing shape patterns that existed at the time for each subspecies. This limitation is further aggravated by the circumstance that Ruttner’s collection was assembled over 50 years ago, and wing venation patterns can change through time, as recently reported for Romanian populations [41].

The detection of wing venation patterns corresponding to the divergent *A. m. ligustica* and *A. m. carnica* in France, Switzerland, the UK, Ireland, Poland, and Russia, therefore mismatching the expected M lineage, can be explained by beekeeper-mediated gene flow and is consistent with molecular surveys reporting variable C-derived introgression in *A. m. mellifera* across Europe [9,10,11,12,13,25,50,54,55,56,57,58,59]. However, *A. m. iberiensis* detected with high probabilities (> 0.950) in colonies located far from the native range in Iberia (French islands, Ireland, the United Kingdom, Switzerland, Sweden, Poland, and Russia) was likely confounded by DeepWings© with its close relative *A. m. mellifera*, as international trading of Iberian queens is very uncommon. If this is true, Switzerland and Groix showed a particularly high rate of misclassification, with 33.3% and 30.6% of the colonies labeled as *A. m. iberiensis,* respectively. In the other locations, the rates were lower (1.9%–20.0%), but still higher than expected, considering that DeepWings© classification accuracy reported for *A. m. mellifera* was 0.950% [44]. This finding calls for an improvement of the software to increase its discriminating power, which implies expansion of the reference database used for training the current version of DeepWings© with wings from collections other than that of Ruttner. DeepWings© is a dynamic tool that can be easily upgraded to include more wings of each subspecies and/or more subspecies by adding their images to retrain a classification model using machine learning [44].

In contrast to the findings north of the Pyrenees, only a small proportion of the colonies (34 from Spain and 3 from Portugal, 5.7%) examined in the *A. m. iberiensis* native range were recognized as *A. m. mellifera* with probabilities above 0.950, indicating that DeepWings© performed relatively well. While misclassification of these colonies cannot be ruled out, the detection of wing shapes matching *A. m. mellifera* can also be explained by the clinal patterns of variation that were recurrently reported for Iberian populations, with populations from northern Spain being genetically closer to *A. m. mellifera* than populations from southern Spain and Portugal [27,28,31,60,61]. However, the six colonies (0.92%) with wing shapes closer to the foreign lineages (although with low probabilities) than to the endemic M lineage were likely misidentified, as suggested by the molecular data (Figure 3). Notably, while the membership proportions inferred from the molecular marker were nearly invariable and above 0.94, the probabilities inferred from the wing marker were scattered along the Y-axis in Figure 3. The disagreement between the two markers does not necessarily imply that the observed wing variation originates from a classification artefact. Since the colonies analyzed here were sampled across three north–south transects, therefore covering the entire native range of *A. m. iberiensis*, it is possible that the probability data reflects genuine variation in the wing venation [28].

In the Azores, where *A. m. iberiensis* was introduced in the XVI century [62], a higher proportion of colonies was assigned to the C-lineage, as compared to Iberia. This finding was particularly noticeable for Graciosa, where most of the colonies were assigned to the C-lineage, and is compatible with high introgression levels obtained from mtDNA markers [47]. Recurrent importations of foreign queens to sustain a breeding program run in the 1980s and 1990s can explain the results of the Azores [47]. Remarkably, this breeding effort left a strong signature on the genetic makeup of all honey bee populations, except on those from São Miguel and Santa Maria. Unlike the populations of Faial, Graciosa, São Jorge, Pico, and Flores, which showed wide variation in both markers, populations from São Miguel and, in particular, from Santa Maria closely resembled Iberian honey bees. While foreign alleles could be purged by genetic drift, it is also possible that selection acted to restore in these two easternmost islands the gene pool that was historically introduced from mainland Iberia.

Similar to the Iberian wings, most Polish wings classified by DeepWings© originate from north–south sampling transects [58]. Yet, the results could not be more divergent between the two areas of the native range of M-lineage. While in Iberia nearly all the colonies matched the endemic subspecies, in Poland, DeepWings© detected a strong diversification of lineages and subspecies. Over 67.1% of the colonies had wing venation patterns more similar to the C-lineage *A. m. carnica* and *A. m. ligustica*, to the O-lineage *A. m. caucasia*, and to the M-lineage *A. m. iberiensis* than to *A. m. mellifera*. While *A. m. iberiensis* could be confounded with *A. m. mellifera*, detection of the other three subspecies is compatible with the existence of a natural hybrid zone in southern Poland, where the three lineages come together [49], as well as with long-standing importations of foreign queens [34,63]. However, detection of a high proportion of wings assigned with high probability to *A. m. ligustica* was unexpected, as molecular studies largely reported in Poland the presence of *A. m. carnica,* but not of the Italian bee [39,58]. Given that C-lineage subspecies are closely related, and therefore, difficult to differentiate, even by molecular markers [17], it is possible that DeepWings© is swapping the two subspecies, as is likely happening with *A. m. mellifera* and *A. m. iberiensis*. Alternatively, but not mutually exclusive, *A. m. ligustica* genes can were introduced in Poland by undocumented importations of Italian queens and/or by the documented and steadily increasing importations of the artificial strain Buckfast [63]. This is a plausible hypothesis because, contrary to Poland, in Croatia, Hungary, Romania, and Slovenia the proportions of wings matching *A. m. ligustica* were low, as would be expected in a territory where *A. m. carnica* is endemic and favored by local beekeepers [5]. Moreover, this finding is consistent with the presence in these countries of mitochondrial and nuclear alleles of *A. m. ligustica* ancestry [29,33,64,65,66,67,68]. Another possibility is that wing images belonging to C-lineage subspecies unrepresented in the DeepWings© reference database and not included in the five-subspecies classifier (e.g., *A. m. cecropia* and *A. m. macedonica*) were identified as *A. m. ligustica*. This could very well be the case of several colonies sampled east and south of the Carpathian mountain ridge in Romania and Moldova, where *A. m. macedonica* and *A. m. carpatica* occurs naturally [5,64,67], that were assigned with low probabilities to *A. m. ligustica*, *A. m. caucasia*, and even to the other Romania-native subspecies *A. m. carnica*.

The association between wing and molecular data, assessed for the colonies sampled in the M-lineage native range, was highly significant but not very strong. Convergence of the two markers was variable and dependent on the integrity of the gene pools. They largely agreed in Iberia, Groix, Ouessant, Santa Maria, São Miguel, Ireland, and Russia, which are known for harboring honey bee populations with high genetic integrity [27,28,47,60,61,69,70,71,72,73,74]. Yet, they often disagreed in the areas where the M-lineage gene pool was threatened by a history of importations of foreign queens, such as in France, UK, or the Azores [9,12,15,47,50,54]. Previous research found that morphological and molecular markers can produce congruent results, supporting the validity of morphometric methods [38,39,75,76]. However, morphometric methods have limitations, especially when dealing with hybridized populations [39,77]. In these populations, the larger variation range of the markers and their overlapping distribution may account for a decrease in the resolution power of the morphometric methods.

While the genetic basis of wing venation is unknown, it is possible that wing traits are encoded by a few genes. Hence, wing markers likely cover a limited portion of the genome variation. In contrast, molecular markers, such as microsatellites and especially SNPs, are widespread across the honey bee genome [20,78], and molecular assays can be designed to fully cover the 16 chromosomes [18,19,24]. If the purpose of colony analysis is to determine the degree of genetic integrity and to estimate introgression proportions with high accuracy, then molecular markers are preferred over wing markers. Otherwise, DeepWings© offers a good alternative for colony identification. By processing batches of up to 40 wings, the software averages out intra-colony variation from a large number of sampled workers, therefore enabling a more robust colony classification. Given that numerous images can be easily and rapidly processed at no cost, DeepWings© is a valuable tool for colony screening in honey bee breeding programs for conservation or other purposes that do not require or do not have a budget for molecular identification.

## Figures and Tables

**Table 1:** Percentages of wings/colonies classified by DeepWings© into each one of the five subspecies according to the origin of the samples (native ranges of *A. m. iberiensis*, *A. m. mellifera,* and *A. m. carnica*). Percentages are shown for the top two classification probabilities and considering a probability threshold > 0.950.

	*A. m. iberiensis* Native Range			*A. m. mellifera* Native Range			*A. m. carnica* Native Range		
Subspecies	1st Highest Probability	2nd Highest Probability	Probability > 0.950	1st Highest Probability	2nd Highest Probability	Probability > 0.950	1st Highest Probability	2nd Highest Probability	Probability > 0.950
*A. m. iberiensis*	77.2/89.7	11.2/5.8	75.0/88.4	11.3/5.8	45.0/31.8	6.7/3.0	1.6/0.0	9.8/3.0	0.5/0.0
*A. m. mellifera*	9.8/4.0	58.8/76.3	19.2/9.6	67.1/41.1	12.8/12.2	80.0/51.4	4.5/1,9	15.1/5.4	1.9/0.4
*A. m. ligustica*	6.8/4.7	10.98.3	3.5/1.5	6.5/12.5	16.5/28.1	3.1/6.0	20.0/9.2	48.9/77.2	10.6/2.2
*A. m. carnica*	3.8/1.0	7.7/3.8	1.9/0.5	11.5/35.9	7.0/12.9	9.3/38.7	72.0/88.3	17.9/9.8	86.7/97.3
*A. m. caucasia*	2.4/0.5	11.4/5.9	0.4/0.0	3.7/4.8	18.7/15.0	0.8/0.9	1.8/0.5	8.3/4.6	0.3/0.0

## Data Availability

The data presented in this study are available on request from the corresponding author.

## Supplementary Materials

The following supporting information can be downloaded at: https://www.mdpi.com/article/10.3390/insects13121132/s1, Table S1: Sampling locations, number of analyzed wings and colonies, and number of loci per molecular marker. Locations highlighted in bold indicate samples originating from conservation programs.; Table S2: Number (N) and probability (median, IQR) of wings classified into each subspecies per dataset., Table S3: Number (N) and probability (median, IQR) of colonies classified into each subspecies per dataset. Figure S1: Accepted and rejected wing images by DeepWings©. (a) Examples of accepted images along with quality parameters. (b) Examples of rejected images. The donut chart represents the percentage of corrupted images distributed across the different classes [79,80].
