# Supplementary material for: Using the Software DeepWings© to Classify Honey Bees across Europe through Wing Geometric Morphometrics
Source: Insects. 2022 Dec 8;13(12):1132. doi: 10.3390/insects13121132 (PMC9784756; doi:10.3390/insects13121132)
Supplement: Supplementary file 1 [file insects-13-01132-s001.zip › insects-2041411-supplementary.pdf]

Table S1. Sampling locations, number of analyzed wings and colonies, and number of loci per molecular marker. Locations highlighted in bold indicate samples originating from conservation programs.

**Country**	**Region**	**Number of wings**	**Number of colonies**	**Number of loci/number of colonies**
Portugal (continent)	North-south Atlantic transect[1]	690	138	383 SNPs[2]/138
Spain	North-South Central and Mediterranean transects[1]	2552	513	383 SNPs[2]/508
Portugal (Azores)	Faial[3]	299	60	73 SNPs[4]/448
	Flores[3]	240	48	
	Graciosa[3]	95	19	
	Pico[3]	366	74	
	Santa Maria[3]	247	50	
	São Jorge[3]	107	26	
	São Miguel[3]	529	106	
	Terceira[3]	386	78	
Belgium	**Limburg (Bosland)**	77	2	
France	Avignon	192	19	91 SNPs[5]/19
	**Groix**	36	36*	13 microsatellites[6]/36
	**Ouessant**	110	11	91 SNPs[5]/11
Ireland	Free living colonies across the country	494	50	12 microsatellites[7]/29
Poland	Collected from flowers across the country	899	899**	
Russia		1377	52	9 microsatellites[8]/8
**Sweden**		549	19	
Switzerland		355	9	
UK	Wales	170	17	107 SNPs/17
Croatia[9]		3048	159	
Hungary	Partially collected from flowers across the country	207	95**	
Moldova		108	10	
Romania[10]		1266	90	
Slovenia		417	21	
Total		14816	2601	

Sampling locations and/or molecular classification in: [1] Chávez-Galarza (2013), [2] Chavez-Galarza (2015), [3] Ferreira (2020), [4]Henriques (manuscript in preparation), [5]Henriques (2021), [6]Garnery (2018), [7]Browne (2020), [8]Ilyasov (2020), [9]Puškadija (2020), [10] Tofilski (2021)

*Each colony was represented by a single wing.

**Samples were collected from flowers at least three kilometers apart, so they can be considered representative of individual colonies. Each colony is represented by a single forewing.

Table S2. Number (N) and probability (median, IQR) of wings classified into each subspecies per dataset.

Country	Region		Subspecies				
			A. m. iberiensis	*A. m. mellifera*	*A. m. ligustica*	*A. m. carnica*	*A. m. caucasia*
Portugal (continent)		N	567	62	20	17	13
		Median, IQR	0.925, 0.233	0.998, 0.009	0.717, 0.421	64.32, 0.310	0.557, 0.257
Spain		N	1975	394	46	49	39
		Median, IQR	0.928, 0.251	0.999, 0.016	0.593, 0.486	0.731, 0.430	0.709, 0.493
Portugal (Azores)	Faial	N	214	9	37	23	3
		Median, IQR	0.910, 0.247	0.869, 0.276	0.737, 0.306	0.731, 0.325	0.654, 0.402
	Flores	N	175	12	19	18	9
		Median, IQR	0.876, 0.319	0.923, 0.157	0.822, 0.291	0.899, 0.163	0.527, 0.323
	Graciosa	N	24	5	48	17	1
		Median, IQR	0.716, 0.247	0.984, 0.026	0.935, 0.246	0.803, 0.320	0.665
	Pico	N	238	19	45	41	6
		Median, IQR	0.884, 0.280	0.989, 0.233	0.768, 0.316	0.776, 0.356	0.540, 0.436
	Santa Maria	N	210	6	1	1	22
		Median, IQR	0.887, 0.262	0.999, 0.122	0.605	0.697	0.654, 0.324
	São Jorge	N	77	1	13	10	4
		Median, IQR	0.920, 0.261	0.938	0.895, 0.220	0.798, 0.271	0.787, 0.450
	São Miguel	N	454	10	28	8	23
		Median, IQR	0.894, 0.243	0.995, 0.076	0.740, 0.324	0.800, 0.460	0.825, 0.262
	Terceira	N	231	9	111	21	9
		Median, IQR	0.818, 0.286	0.963, 0.107	0.827, 0.356	0.777, 0.347	0.563, 0.334
Belgium		N	11	62		1	
		Median, IQR	0.964, 0.278	1.000, 0.002		0.994	
France	Avignon	N	20	30	42	38	16
		Median, IQR	0.828, 0.322	0.975, 0.378	0.901, 0.235	0.954, 0.306	0.881, 0.379
	Groix	N	11	23	2		
		Median, IQR	0.979, 0.196	0.996, 0.088	0.643, 0.096		
	Ouessant	N	13	47		4	3
		Median, IQR	0.972, 0.351	1.000, 0.010		0.896, 0.406	0.875, 0.336
Ireland		N	73	316	9	3	4
		Median, IQR	0.975, 0.108	1.000, 0.002	1.000, 0.195	1.000, 0.004	0.785, 0.359
Poland		N	24	261	112	353	43
		Median, IQR	0.638, 0.441	0.975, 0.169	0.855, 0.309	0.983, 0.148	0.641, 0.463

Russia		N	96	1214	9	9	14
		Median, IQR	0.843, 0.314	1.000, 0.005	0.798, 0.190	0.978, 0.419	0.791, 0.450
Sweden		N	92	414	10		
		Median, IQR	0.941, 0.285	1.000, 0.004	1.000, 0.433		
Switzerland		N	72	158	53	18	27
		Median, IQR	0.910, 0.269	0.995, 0.044	0.932, 0.259	0.792, 0.452	0.760, 0.335
UK	Wales	N	20	48	12	15	33
		Median, IQR	0.609, 0.522	0.965, 0.167	0.755, 0.375	0.798, 0.421	0.792, 0.344
Croatia		N	35	107	590	2142	33
		Median, IQR	0.645, 0.341	0.860, 0.349	0.856, 0.303	0.991, 0.085	0.639, 0.314
Hungary		N	4	11	26	151	
		Median, IQR	0.866, 0.167	0.764, 0.234	0.830, 0.378	0.996, 0.056	
Moldova		N	11	10	28	36	18
		Median, IQR	0.547, 0.376	0.711, 0.273	0.859, 0.286	0.972, 0.171	0.725, 0.333
Romania		N	23	69	238	782	33
		Median, IQR	0.800, 0.397	0.793, 0.346	0.873, 0.276	0.990, 0.099	0.697, 0.343
Slovenia		N	3	16	65	297	3
		Median, IQR	0.868, 0.648	0.711, 0.423	0.808, 0.353	0.995, 0.060	0.578, 0.189
Total		N	4673	3313	1564	4054	356
		Median, IQR	0.908, 0.265	0.999, 0.023	0.0847, 0.309	0.988, 0.111	0.702, 0.339

Table S3. Number (N) and probability (media, IQR) of colonies classified into each subspecies per dataset.

Country	Region		Subspecies				
			A. m. iberiensis	*A. m. mellifera*	*A. m. ligustica*	*A. m. carnica*	*A. m. caucasia*
Portugal (continent)		N	132	4	2		
		Median, IQR	0.935, 0.210	0.976, 0.141	0.550, 0.232		
Spain		N	471	38	1	1	2
		Median, IQR	0.918, 0.226	0.998, 0.005	0.764	0.994	0.520, 0.172
Portugal (Azores)	Faial	N	57	1	2		
		Median, IQR	0.924, 0.243	0.922	0.716, 0.564		
	Flores	N	44	1	2	1	
		Median, IQR	0.806, 0.313	0.681	0.638, 0.086	0.886	
	Graciosa	N	4		14	1	
		Median, IQR	0.740, 0.388		0.801, 0.349	0.525	
	Pico	N	67		4	2	1
		Median, IQR	0.881, 0.260		0.637, 0.280	0.718, 0.315	0.602
	Santa Maria	N	49				1
		Median, IQR	0.769, 0.318				0.551
	São Jorge	N	23		1	2	
		Median, IQR	0.953, 0.210		0.927	0.608, 0.598	
	São Miguel	N	101	1		2	2
		Median, IQR	0.812, 0.336	0.994		0.869, 0.222	0.452, 0.002
	Terceira	N	50		26	2	
		Median, IQR	0.695, 0.311		0.679, 0.288	0.538, 0.153	
Belgium		N		2			
		Median, IQR		1.000, 0.000			
France	Avignon	N	4	3	6	5	1
		Median, IQR	0.747, 0.374	0.990, 0.289	0.864, 0.388	0.992, 0.388	0.805
	Groix	N	11	23	2		
		Median, IQR	0.988, 0.196	0.996, 0.088	0.643, 0.096		
	Ouessant	N	2	8		1	
		Median, IQR	0.992, 0.016	0.998, 0.032		97.99	
Ireland		N	7	39	2	2	

		Median, IQR	0.859, 0.279	1.000, 0.002	1.000, 0.001	0.997, 0.005	
Poland		N	24	261	112	353	43
		Median, IQR	0.637, 0.255	0.975, 0.167	0.855, 0.268	0.983, 0.148	0.641, 0.316
Russia		N	1	50	1		
		Median, IQR	0.571	0.998, 0.002	0.537		
Sweden		N	3	16			
		Median, IQR	0.850, 0.436	0.999, 0.016			
Switzerland		N	3	5	1		
		Median, IQR	0.924, 0.126	0.992, 0.018	0.718		
UK	Wales	N	3	7	2	1	4
		Median, IQR	0.816, 0.462	0.758, 0.337	0.756, 0.2787	0.994	0.770, 0.189
Croatia		N		1	14	144	
		Median, IQR		0.704, 0.000	0.664, 0.248	0.983, 0.058	
Hungary		N		5	9	74	
		Median, IQR		0.790, 0.240	0.922, 0.122	0.982, 0.121	
Moldova		N			3	5	2
		Median, IQR			0.787, 0.341	0.695, 0.471	0.600, 0.096
Romania		N		1	7	82	
		Median, IQR		0.624, 0.000	0.815, 0.252	0.989, 0.117	
Slovenia		N			1	20	
		Median, IQR			0.752, 0.000	0.993, 0.040	
Total		N	1056	466	212	698	56
		Median, IQR	0.887, 0.275	0.994, 0.075	0.800, 0.290	0.983, 0.119	0.636, 0.334

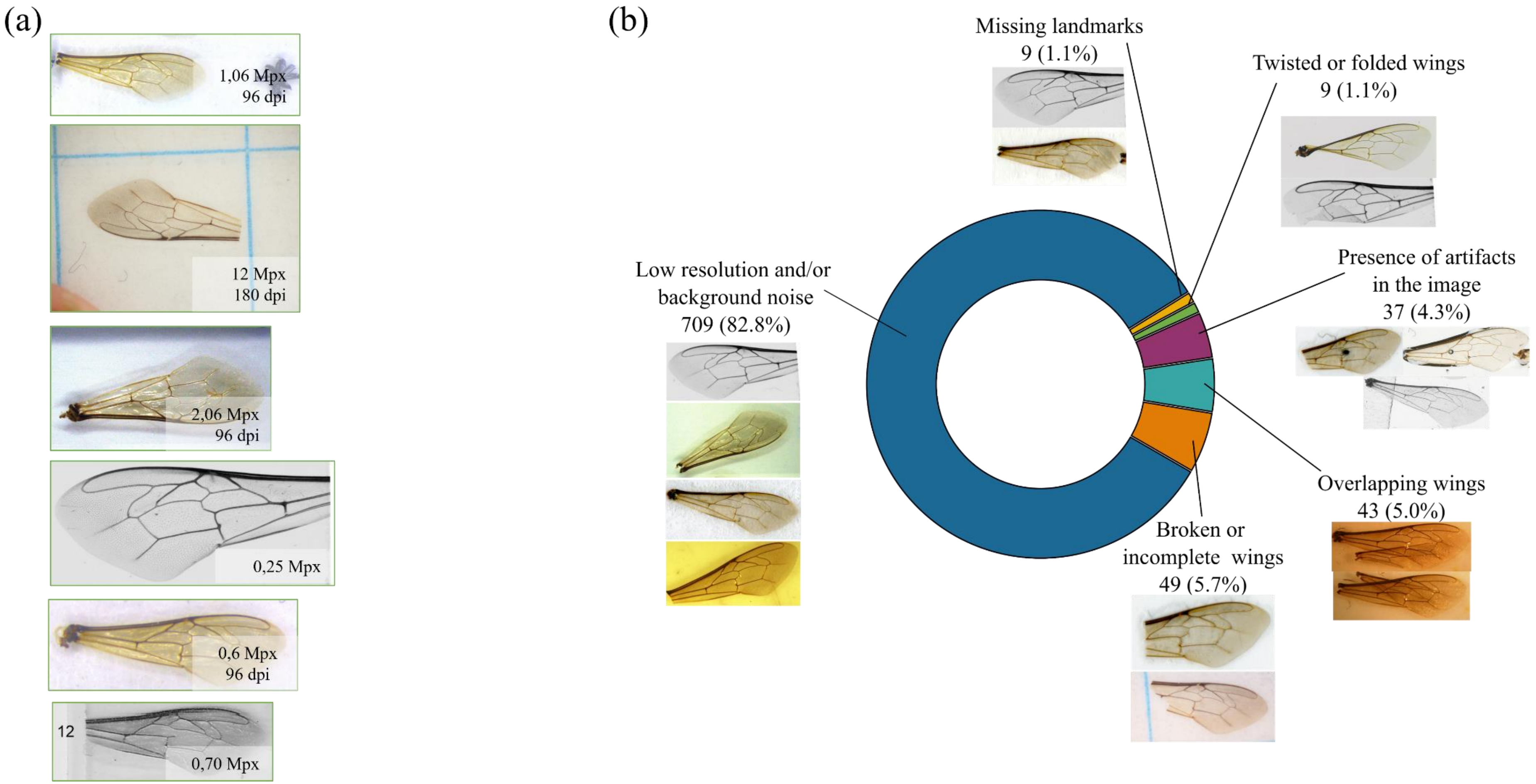

Figure S1. Accepted and rejected wing images by DeepWings©. (a) Examples of accepted images along with quality parameters. (b) Examples of rejected images. The donut chart represents the percentage of corrupted images distributed across the different classes.

References

1. Chávez-Galarza, J.; Henriques, D.; Johnston, J.S.; Azevedo, J.C.; Patton, J.C.; Muñoz, I.; De la Rúa, P.; Pinto, M.A. Signatures of selection in the Iberian honey bee (*Apis mellifera iberiensis*) revealed by a genome scan analysis of single nucleotide polymorphisms. *Mol. Ecol.* **2013**, *22*, 5890–5907.
2. Chávez-Galarza, J.; Henriques, D.; Johnston, J.S.; Carneiro, M.; Rufino, J.; Patton, J.C.; Pinto, M.A. Revisiting the Iberian honey bee (*Apis mellifera iberiensis*) contact zone: maternal and genome-wide nuclear variations provide support for secondary contact from historical refugia. *Mol. Ecol.* **2015**, *24*, 2973–2992.
3. Ferreira, H.; Henriques, D.; Neves, C.J.; Machado, C.A.; Azevedo, J.C.; Francoy, T.M.; Pinto, M.A. Historical and contemporaneous human-mediated processes left a strong genetic signature on honey bee populations from the Macaronesian archipelago of the Azores. *Apidologie* **2020**, *51*, 316–328.
4. Henriques, D.; Lopes, A.R.; Pinto, M.A. Centro de Investigaçao de Montanha-Instituto Politécnico de Bragança, Bragança, Portugal. *Manuscript in preparation*.
5. Henriques, D.; Lopes, A.R.; Chejanovsky, N.; Dalmon, A.; Higes, M.; Jabal-Uriel, C.; Le Conte, Y.; Reyes-Carreño, M.; Soroker, V.; Martín-Hernández, R. Mitochondrial and nuclear diversity of colonies of varying origins: contrasting patterns inferred from the intergenic tRNAleu-cox2 region and immune SNPs. *J. Apic. Res.* **2021**, *61*, 305–308.
6. Garnery, L. *Rapport d´ Expertise 2018. Analyses Génétiques de la Population d´Abeilles Melliféres de I´Ile de Groix*; Laboratoires Evolution Génomes Comportement et Ecologie (Gif-sur-Yvette, France) 2018.
7. Browne, K.A.; Hassett, J.; Geary, M.; Moore, E.; Henriques, D.; Soland-Reckeweg, G.; Ferrari, R.; Mac Loughlin, E.; O'Brien, E.; O'Driscoll, S. Investigation of free-living honey bee colonies in Ireland. *J. Apic. Res.* **2020**, *60*, 229–240.
8. Ilyasov, R.A.; Lee, M.-L.; Yunusbaev, U.; Nikolenko, A.; Kwon, H.-W. Estimation of C-derived introgression into A. m. mellifera colonies in the Russian Urals using microsatellite genotyping. *Genes Genom.* **2020**, *42*, 987–996.
9. Puškadija, Z.; Kovačić, M.; Raguž, N.; Lukić, B.; Prešern, J.; Tofilski, A. Morphological diversity of Carniolan honey bee (Apis mellifera carnica) in Croatia and Slovenia. *J. Apic. Res.* **2020**, *60*, 326–336.
10. Tofilski, A.; Căuia, E.; Siceanu, A.; Vișan, G.O.; Căuia, D. Historical Changes in Honey Bee Wing Venation in Romania. *Insects* **2021**, *12*, 542.
